# Salmonella Typhimurium Myocarditis in Two Previously Healthy Children

**DOI:** 10.7759/cureus.62135

**Published:** 2024-06-11

**Authors:** Fani Ladomenou, Ekaterini Nikolaou, Marianna Deligeorgopoulou, Konstantina Kapetaniou, Antonios Vlahos, Ekaterini Siomou

**Affiliations:** 1 Department of Paediatrics, University Hospital of Ioannina, Ioannina, GRC

**Keywords:** child, diarrhea, salmonella typhimurium, non-typhoidal salmonella, myocarditis

## Abstract

Myocarditis represents an inflammation affecting the heart muscles, a condition relatively uncommon among children. Its diagnosis poses challenges due to the diverse range of its non-specific symptoms. Non-typhoidal *Salmonella *(NTS) species are known as rare but noteworthy contributors to myocarditis, especially among immunocompetent young patients. We present two cases of NTS myocarditis in previously healthy children, in an attempt to shed light on the epidemiology, diagnostic methods, and prognosis, aiming to offer a greater understanding of this rare condition.

## Introduction

Myocarditis represents an inflammation affecting the heart muscles, a condition relatively uncommon among children, estimated to occur at a rate of one to two cases per 100000 children annually [1]. Its diagnosis poses challenges due to the diverse range of its non-specific symptoms. Studies underline myocarditis as the third leading cause of mortality in young athletes [2]. If left unattended, this inflammatory process may progress to acute cardiac failure and persistent dilated cardiomyopathy. While the specific origin remains elusive, factors such as increased inflammation, susceptibility to heart failure, and markers like testosterone, innate immune components, and pro-fibrotic cytokines have been thought of as potential contributors [3]. Viral infections, particularly Coxsackie and adenovirus, are prevalent in the UK and Europe, whereas bacterial infections (such as Diphtheria, Legionella, Mycobacterium Tuberculosis, and Salmonella), albeit rare, are noteworthy causes of myocarditis [3]. Although endo-myocardial biopsy is considered the gold standard for diagnosis, there is a growing preference for non-invasive techniques due to their reduced risk for complications. Monitoring troponin levels continuously is crucial, especially when myocarditis leads to a gradual rise in troponin levels within a 24-hour span, peaking around day two or three after disease onset. Among diagnostic methodologies, cardiac magnetic resonance imaging (CMRI) has emerged as the primary diagnostic tool due to its safety and accuracy. It is noteworthy that MRI will detect lesions of myocarditis but will not contribute to identifying the pathogen [2].

Salmonellosis is a bacterial infection known for causing severe illness and fatalities worldwide. While Salmonella infections result in various clinical manifestations, they are an uncommon cause of myocarditis, especially among immunocompetent young individuals [2]. We describe two cases of non-typhoidal Salmonella (NTS) myocarditis, with the aim of shedding light on its epidemiology, diagnostic methods, and prognosis, offering a better understanding of this rare condition.

## Case presentation

Case 1

An eight-year-old previously healthy boy was transferred to our tertiary hospital's emergency department after hospitalization for five days in a district hospital. The patient presented with a history of high fever lasting seven days, vomiting, and watery non-bloody diarrhea accompanied by severe abdominal cramps. Laboratory evaluation revealed elevated levels of C-reactive protein (CRP) at 56.6 mg/L (normal range: <5 mg/L), while a stool culture grew *Salmonella enterica* serovar Typhimurium (S. enterica ser. Typhimurium) using antigenic analysis of O and H antigens (White Kauffmann-Le Minor Scheme formulae). The blood culture showed no growth. The initial management included intravenous fluids and pain relief.

A day before admission to our department, despite being afebrile for 48 hours but with persistent gastrointestinal symptoms, the patient experienced sudden chest pain of 30-40 minutes duration, described as a retrosternal "squeezing" sensation without any specific triggers or relieving factors. The pain resolved spontaneously. While in pain, his heart rate was 140/minute (normal range: 70-110 bpm), and his blood pressure was 102/68 mmHg (95th percentile for age, sex, and height: 114/74 mmHg). The rest of the physical examination was unremarkable. Laboratory investigations revealed a normal white blood cell count (10460/mL, normal range up to 11000/mL) and subsequently a normal CRP level (4.9 mg/L, normal range up to 5 mg/L). The initial troponin I concentration was significantly high at 1010 ng/mL, which increased to 525.1 ng/mL after 24 hours (Table 1) [4]. Echocardiography showed a normal left ventricular function.

**Table 1:** hs-Troponin levels of Case 1 and Case 2.

Days	Case 1 (hs Troponin-I ng/ml)	Case 2 (hs Troponin-I ng/ml)	Normal Values (3) (ng/ml)
Day1 (1^st^)	1010	388	0.0001-0.006
Day1 (2^nd^)	525.1	223.2	0.0001-0.006
Day2	387.4	278.1	0.0001-0.006
Day3	223.9	333.7	0.0001-0.006
Day4	20	42.5	0.0001-0.006
Day5	3.1	7.4	0.0001-0.006
Day6	<2.3	18.6	0.0001-0.006

With a working diagnosis of myocarditis triggered by *Salmonella* infection, the patient received broad-spectrum antibiotics intravenous (Cefotaxime 150mg/kg) and fluids. Due to the persistent elevation in troponin levels, he was transferred to our pediatric cardiology department. Upon admission, his vital signs were within normal limits, and the physical examination was unremarkable. The initial electrocardiogram (ECG) was normal. 

Laboratory tests including antigenic tests for herpesviruses (Epstein-Barr virus (EBV) and cytomegalovirus (CMV)) and enteroviruses (Coxsackie, ECHO) and nasopharyngeal PCR for herpesviruses were conducted and yielded negative results. Furthermore, blood and stool cultures were performed. During hospitalization, troponin levels, which peaked at 1010 ng/mL within the first 24 hours, gradually declined to 2.3 ng/mL on the sixth day of his hospitalization. Stool culture confirmed the presence of *Salmonella enterica* serovar Typhimurium, using the White- Kauffmann-Le Minor Scheme antigenic formula. While blood cultures did not reveal any evidence of bacteremia.

**Figure 1:**
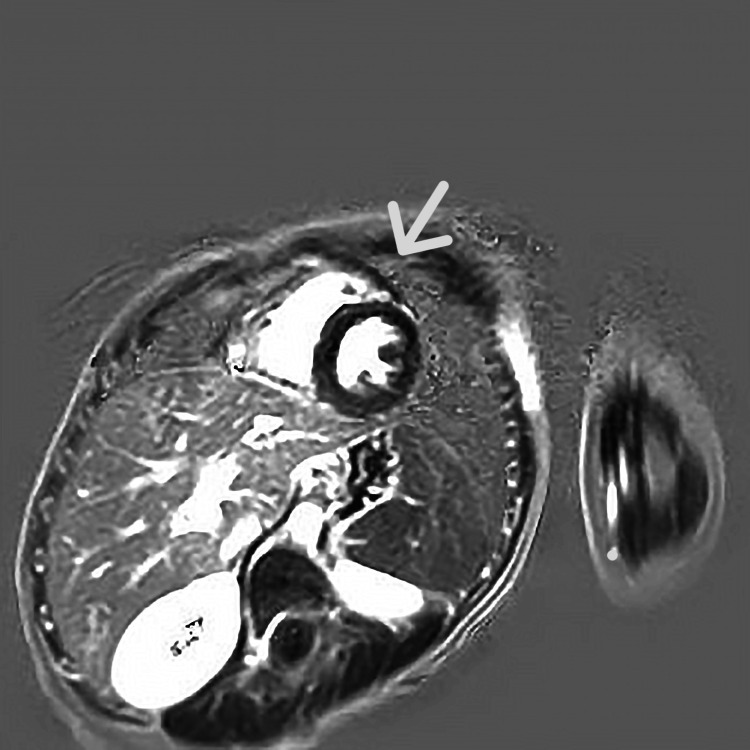
cMRI: Linear hypoechoic enhancement in the basal anterior and basal posterior septal wall of the left ventricle. cMRI: Cardiac magnetic resonance imaging

On the second day, a transthoracic echocardiogram (TTE) showed a normal ejection fraction (EF 77%), no regional wall motion abnormalities, and no pericardial effusion. Cardiac magnetic resonance imaging (cMRI) revealed abnormal sub-epicardial contrast enhancement in multiple areas, indicating multifocal myocarditis. (Figure 1). The patient showed clinical and laboratory improvement over the following days. The ECG was persistently normal. In total, he received antibiotic therapy (intravenous Cefotaxime 150mg/kg) for 10 days. Post-discharge recommendations included avoiding physical exertion during the acute phase of myocarditis, with a potential return to physical activities after six months pending normal findings on a Holter monitor, stress tests, and the continuation of normal ECG results. Given that this examination did not reveal any pathological findings, a repeat cMRI was conducted, which also yielded normal results. Consequently, the patient was able to resume his activities without any restrictions.

Case 2

A five-year-old girl was admitted to our department with a five-day history of fever, watery diarrhea, and elevated troponin levels (388 ng/mL). The girl was initially admitted to a district hospital with a four-day history of fever up to 39.2^o^ C and watery, non-bloody diarrhea. The laboratory tests did not reveal elevated inflammatory markers (erythrocyte sedimentation rate 3, CRP 6mg/ L) but hs troponin-I levels were checked due to marked tachycardia and were found abnormal at 223.2 ng/mL [4]. Due to the high troponin levels, an echocardiogram was performed which did not reveal any abnormal findings. The patient had been treated with trimethoprim/sulfamethoxazole (10 mg/kg/d trimethoprim) orally for three days prior to hospitalization. During her hospitalization, ampicillin was commenced intravenously at a dose of 200mg/kg/d.

On admission to our department, the girl was in a good general condition. She was not tachycardic or tachypneic. Her blood pressure was 108/56 mmHg (95h centile 128/84 mmHg). Cardiac auscultation revealed mild arrhythmic heart sounds and a systolic murmur, which was due to an already known ventricular septal defect.

Initial laboratory tests showed white blood cells 7670/μl, neutrophils 4111/μl, hemoglobin 10.7g/dL, CRP 6 mg/L, troponin I 223.2 pg/mL, CK 67 IU/L, creatinine 0.59 mg/dL, and urea 20 mg/dL. The ECG demonstrated sinus rhythm, and a T- wave inversion in lead V4 with resolution of the finding on the second day of hospitalization. Stool culture obtained on the second day after admission as well as the stool culture obtained at the district hospital confirmed *Salmonella enterica* serovar Typhimurium (Ampicillin sensitive). Serological testing for EBV, CMV, and enteroviruses was negative. Furthermore, blood cultures did not reveal any pathogen.

**Figure 2:**
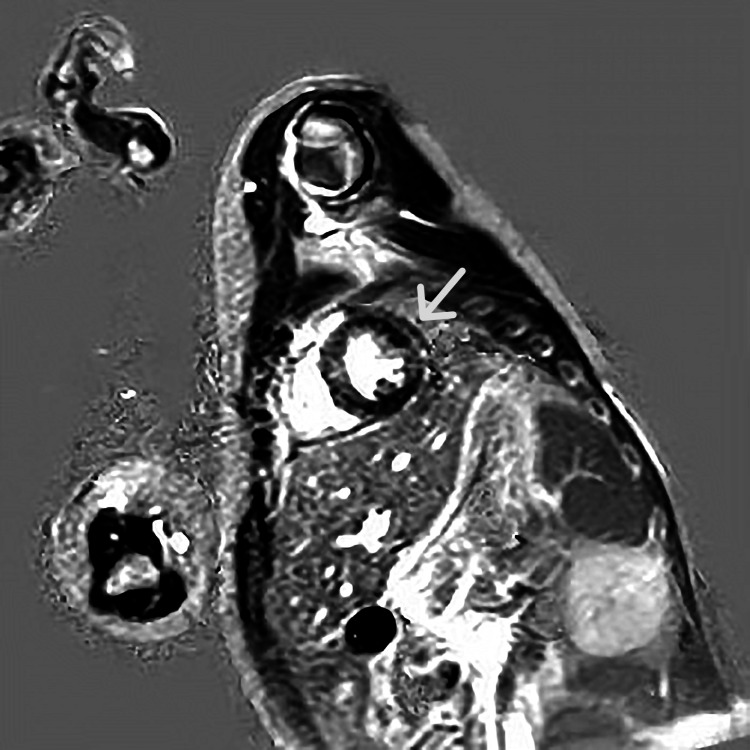
cMRI: Intraventricular septum enhancement. cMRI: Cardiac magnetic resonance imaging

A cMRI was performed on the second day of her admission which showed delayed gadolinium enhancement of the interventricular septum, a finding consistent with myocarditis (Figure 2). Left ventricular contractility was not affected, with an ejection fraction of 65%. Wall hypokinesis or pericardial fluid was not demonstrated. A TTE was obtained which did not reveal any abnormality.

The patient was discharged after a 10-day course of intravenous ampicillin. During her hospital stay, she remained hemodynamically stable and asymptomatic. Troponin levels normalized on the fifth day of her hospitalization (7.4 pg/ml) (Table 1). Post-discharge recommendations included avoiding physical exertion during the acute phase of myocarditis, with a potential return to physical activities after six months pending normal findings on a Holter monitor, stress tests, and the continuation of normal ECG results.

At the two-month follow-up, the girl had completely recovered and the echocardiogram showed no hypokinesis or motion abnormalities.

## Discussion

Myocarditis is an inflammatory condition of the myocardium that poses diagnostic challenges due to its broad spectrum of clinical manifestations, varying from asymptomatic cases to severe cardiovascular events and even heart failure, occasionally leading to life-threatening conditions [5].

Individuals who exhibit mild symptoms or have no symptoms with maintained left ventricular function generally experience a favorable prognosis and a lower risk of complications. Conversely, individuals exhibiting a left ventricular ejection fraction under 50%, ventricular arrhythmias, or reduced cardiac output syndrome tend to experience less favorable outcomes. The prognosis of myocarditis hinges on the underlying cause.

Both infectious and non-infectious etiologies have been implicated in the pathogenesis of myocarditis. Although viral infections (Adenovirus, EBV, CMV, CMV, Coxsackie, Influenza, etc.) are frequently cited as the predominant cause, bacterial infections (Bartonella, Brucella, Diphtheria, Haemophilus, Salmonella, etc.), though less prevalent (ranging from 0.2 to 1.5%), should consistently be under investigation [5]. Additionally, non-infectious elements like cardiotoxins (e.g., alcohol, anthracyclines, or cocaine), hypersensitivity reactions, systemic illnesses (e.g., celiac disease, sarcoidosis, or thyrotoxicosis), and radiation should be duly considered.

Salmonella spp. are Gram (-) bacilli that can be broadly classified into typhoidal and non-typhoidal forms and are responsible for significant morbidity and mortality in both developed and developing countries [6]. Both typhoidal and NTS can have extra-intestinal manifestations. Cardiovascular involvement most frequently manifests as endocarditis, affecting both valvular and non-valvular structures. In rare circumstances, Salmonella can cause myocarditis. Typhoid fever, caused by *Salmonella typhi *and *Salmonella paratyphi*, remains the most prevalent form of Salmonella illness associated with myocarditis. However, myocarditis can also be a rare extra-intestinal manifestation of NTS infection potentially resulting in dilated cardiomyopathy with considerable morbidity and mortality [5].

NTS causes a significant global burden of diarrheal illness, resulting in millions of reported cases and thousands of fatalities annually. Generally, NTS infection is a self-limiting illness associated with foodborne transmission. However, in less than 5% of cases, Salmonella infection can progress to bacteremia, especially in individuals with underlying immune deficiencies, such as those with HIV infection [5,6]. Our patients were both healthy before enteric infection without known immunodeficiency. In predisposed immunosuppressed patients usually, there are positive blood cultures and a fatal outcome [6]. Bacteremia can lead to diverse cardiovascular complications, including endocarditis, pericarditis, vascular infections, or osteomyelitis. Notably, among individuals with pre-existing atherosclerosis, endocarditis stands as the most common cardiovascular complication [7]. In the case of Salmonella myocarditis, the hypothesis is that myocardial damage occurs secondary to involvement of endocardium or due to direct bacterial invasion from bacteremia [6]. In addition to this, sepsis-induced myocardial depression and subsequent remodeling may also play a part as it does in other cases of bacterial myocarditis [6].

A systematic review of NTS myocarditis reported in the literature included 24 individuals (19 adults and five pediatric cases; the mean age for adults and pediatric cases was 36.6 years and 9.6 years respectively) and highlighted fever, abdominal discomfort, and chest pain as the commonest presenting symptoms, consistent with our patients' clinical presentation [6]. Of note, less than 25% of the cases had associated diarrhea. However, both of our patients had persistent gastrointestinal symptoms when myocarditis was diagnosed. Moreover, this review highlighted *S. enteritidis* as the most common etiological agent (40%), with *Salmonella* Typhimurium being the most frequently reported pathogen in the adult group (36.8% of cases) and *S. Enteritidis *being the most frequently reported pathogen in the pediatric group (80% of cases) [6]. To the best of our knowledge, our patients are the first pediatric cases reported in the literature with myocarditis caused by *Salmonella* Typhimurium. Both our patients had an uneventful course with a favorable outcome. The overall mortality reported in the literature is 24% while 42% of patients require intensive care [6].

Although the reports from the literature show a male predominance in *Salmonella* myocarditis in adults, female prevalence has been reported in the pediatric population [7]. While testosterone has been identified as a risk factor for myocarditis, explaining the preponderance in adult males, other factors including immune function and innate defenses likely play a role, as evidenced by the lack of cardiac symptoms in the patient's companion [2].

Our two patients had no pre-existing cardiovascular disease or detectable immune deficiencies, highlighting that even generally healthy individuals might be susceptible to Salmonella myocarditis. Their elevated cardiac enzymes and inflammatory markers, coupled with the detection of *Salmonella *Typhimurium in stool cultures, contributed to the diagnosis, corroborated by CMRI findings.

## Conclusions

*Salmonella *Typhimurium, although a rare case of myocarditis, should always be considered in cases presenting with diarrhea and signs of angina pectoris in the absence of viral etiology. Given that invasive *Salmonella* Typhimurium infections are more prevalent in immunocompromised patients, screening for immune dysfunction may be warranted. While antibiotics are not typically prescribed for Salmonella gastroenteritis, the onset of systemic complications such as myocarditis may require immediate antimicrobial treatment, which could potentially mitigate the chances of enduring effects such as dilated cardiomyopathy.
